# Perception gaps between healthcare professionals and people with CLBP: an online survey of current primary care management practices in the United Kingdom

**DOI:** 10.1080/07853890.2025.2553216

**Published:** 2025-09-17

**Authors:** Tianyu Zhou

**Affiliations:** ^a^ School of Exercise and Health, Shanghai University of Sport, Shanghai, China; ^b^ MSk Lab, Department of Surgery & Cancer, Imperial College London, London, United Kingdom

**Keywords:** Low back pain, biopsychosocial model, patient-centred care, health personnel, surveys and questionnaires, primary health care

## Abstract

**Background:**

This study examined differences in perspectives and perceptual gaps in the management of chronic low back pain (CLBP) between healthcare professionals—general practitioners (GPs) and physiotherapists—and people with CLBP in United Kingdom primary care.

**Methods:**

A cross-sectional online survey was completed by 32 GPs, 53 physiotherapists, and 138 people with CLBP. Closed- and open-ended questions assessed management strategies, perceived effectiveness, treatment goals, and alignment of perspectives. Quantitative data were analysed descriptively and qualitative responses through content analysis.

**Results:**

GPs most frequently recommended exercise (87.5%), analgesics (84.4%), and referral to physiotherapy (62.5%). Physiotherapists emphasised exercise (98%), pain education (73.6%), and active lifestyle advice (47.2%). Both groups prioritised active management, while patients most often reported analgesic use (44.9%) and passive treatments such as manual therapy (12.3%) and electrotherapy (12.3%). Regarding treatment goals, GPs and physiotherapists prioritised functional improvement, pain reduction, mental health, daily activity, and quality of life (all ≥80%), reflecting a biopsychosocial approach. Patients, however, focused mainly on pain relief (52.2%) and return to activity (33.3%), underscoring expectation gaps.

**Conclusions:**

While GPs and physiotherapists generally follow guideline-recommended, active strategies for CLBP, substantial differences remain between clinician recommendations and patient-reported practices and goals. Addressing these gaps requires enhanced communication, shared decision-making, and more person-centred care.

## 1. Introduction

In the UK, chronic low back pain (CLBP) is one of the most common reasons for consultation with a primary care practitioner. The prevalence of medical care-seeking for back pain in primary care was 47% among people under 50 years of age, rising to 64% among people aged over 70 years [1]. People with CLBP typically seek care through the National Health Service (NHS), where general practitioners (GPs) and primary care physiotherapists act as the first point of contact within the healthcare system. Management typically involves advice to remain active, prescription of analgesics, referral to physiotherapy and in some cases, onward referral to pain clinics or musculoskeletal services [2]. Evidence suggests that while clinical guidelines recommend a biopsychosocial approach, including education, physical activity and psychological support, actual practice often leans towards pharmacological or passive modalities, especially in cases of patient demand or time constraints in primary care consultations [3].

Clinical practice guidelines (CPGs) have been developed to help healthcare professionals provide care consistent with evidence-based practice in treating low back pain (LBP) [4]. These guidelines endeavour to locate, review and summarise the best available scientific evidence, and consequently, guidelines are vital tools for clinicians [2,5]. Adherence to recommendations made by guidelines regarding CLBP has been linked to both improved clinical outcomes and decreased costs [6]. The 2020 NICE guideline provides evidence-based back pain recommendations to assist practitioners’ decisions about appropriate healthcare provision in the UK [7]. Although progress has been made in developing and updating evidence-based guidelines, such knowledge needs to be translated into clinical practice [8]. This can be complex and will be influenced by a range of factors, including the availability of resources within the healthcare system, the knowledge and experience of the clinician and, indeed, the socioeconomic status of the patient [9]. This means that the translation of guidelines into clinical practice is often suboptimal [10,11]. However, the extent to which UK primary care practices reflect the recommendations of the guidelines and the effectiveness of those practices is unclear. A previous study identified the content of advice given by clinicians to people with back pain in a UK primary care clinical setting [12]. Limited research has surveyed the management strategies for CLBP within UK primary care settings from both the perspectives of clinicians and patients, as well as the perceived effectiveness of these treatments and the treatment outcomes that are important for CLBP.

A cross-sectional survey was therefore designed to capture a snapshot of current clinical practice and the lived experiences of people with CLBP—including management strategies, perceived treatment effectiveness and important outcomes. Therefore, this study aimed to address the following questions:

- What advice and treatments do healthcare professionals working in primary care provide for CLBP?
- How do people with CLBP describe the treatment that they have been given for their back pain?
- How effective are interventions offered in primary care practice perceived to be by people with back pain?
- What are the important outcomes for people with CLBP?

## 2. Methods

### 2.1. Study design

This study adopted a cross-sectional mixed-methods design, utilising an online survey comprising both closed-ended and open-ended questions to explore the views of primary care clinicians and people with CLBP regarding its management. The closed-ended questions were used to quantify characteristics and preferences among participants, providing clear statistical data. The open-ended questions were included to capture deeper, qualitative insights into participants’ experiences and perspectives, offering a richer understanding of their attitudes and beliefs [13]. The qualitative data were analysed using an inductive content analysis approach, allowing key themes to emerge from the data without pre-defined categories. Online surveys were chosen for their accessibility and convenience, allowing participation from primary care clinicians and people with CLBP across diverse geographical locations.

This study follows the STROBE (Strengthening the Reporting of Observational Studies in Epidemiology) guidelines for observational studies [14] and the CHERRIES (Checklist for Reporting Results of Internet E-Surveys) guidelines for online survey reporting [15]. The completed checklists for both STROBE and CHERRIES are provided in the Supplementary Appendix A and Supplementary Appendix B. Ethical approval for this study was obtained from the Imperial College Research Ethics Committee (ICREC) at Imperial College London (Reference number: 6271213). All procedures involving human participants were conducted in accordance with the ethical standards of the committee and with the 1964 Declaration of Helsinki and its later amendments or comparable ethical standards.

### 2.2. Recruitment

Primary care clinicians were recruited through targeted dissemination of the study recruitment poster *via* their private social media groups (e.g. WhatsApp), email lists and professional community networks. Additionally, the recruitment poster was uploaded and shared on the official websites and communication channels of professional bodies, including The Royal College of General Practitioners, Primary Care Rheumatology and Musculoskeletal Medicine Society and the Chartered Society of Physiotherapy, facilitating wider reach among clinicians.

People with experience of CLBP were recruited by sharing the recruitment poster within patient-focused social media groups and communities (e.g. WhatsApp groups, My Neighbourhood app, email chains and Twitter). All participants were also encouraged to forward the invitation to relevant clinicians and individuals with CLBP to maximize reach.

As this was a cross-sectional study employing convenience sampling, no formal sample size calculation was conducted, and there was no predetermined target sample size.

### 2.3. Eligibility criteria

Criteria for inclusion in the review were: (1) Aged 18 years of age or older, (2) People with experience of LBP lasting for 3 months or more; or (3) Primary care healthcare professionals, specifically GPs or physiotherapists, with experience in managing people with CLBP in a primary care setting. Exclusion criteria were: (1) Under 18 years old, (2) People with experience of LBP less than 3 months, (3) People with LBP attributed to specific diagnosed pathologies, such as radiculopathy, spinal fractures, infections, malignancies, or other identifiable causes.

### 2.4. Questionnaire design

A cross-sectional, web-based questionnaire was developed for two groups: healthcare professionals in UK primary care and individuals with experience of CLBP. Both questionnaires contained a mix of open-ended and closed-ended questions, covering demographic information and CLBP-related experiences.

The healthcare professional’s questionnaire collected data on professional role, years of experience, approaches to advising and treating people with CLBP, perceived treatment effectiveness, delivery of existing care packages and outcomes considered most important for people with CLBP (Supplementary Appendix C). The patient questionnaire gathered information on back pain duration, treatments received, perceived treatment effectiveness, preferred treatment approaches and indicators of improvement (Supplementary Appendix D). Both questionnaires were reviewed by representatives of the target group (healthcare professionals or people with CLBP) to ensure clarity, relevance and usability; minor wording changes were made based on feedback.

**Questionnaire for healthcare professionals**

- *Closed-ended questions (Q1–Q4)*
- What is your age?
- What is your gender?
- What is your role in primary care?
- How many years have you been in this role?
- *Open-ended questions (Q5–Q8)*
- What advice do you typically provide to patients with CLBP and how is this advice delivered?
- What treatments do you typically provide to patients with CLBP, and how are they delivered?
- Do you think the advice and treatment you give to patients is effective? Please let us know why.
- What outcomes do you think are important to CLBP patients? Please specify why you select this option.

**Questionnaire for people with CLBP**

- *Closed-ended questions (Q1–Q4)*
- What is your age?
- What is your gender?
- What is your race?
- How long have you had your back pain?
- *Open-ended questions (Q5–Q8)*
- What treatment have you been given for your back pain?
- Do you think the treatment helped? Can you tell us why?
- What sort of treatment do you think would help your back pain?
- What things would help you know that you were getting better? Please let us know why.

### 2.5. Data collection

The survey was posted between January and March 2024 using the strategy outlined above. Qualtrics survey software (Qualtrics, Provo, Utah, USA) was used to disseminate the survey and collect data. The online survey took approximately 15 min to complete. To maximise reach, the survey invitation was periodically re-shared on social media and professional platforms during the recruitment period. At the start of the questionnaire, participants were provided with written information outlining the purpose, scope, procedures and data storage of the study. Written informed consent was obtained electronically before participation by asking participants to confirm their consent *via* a mandatory checkbox before proceeding with the questionnaire. Participation was entirely voluntary, with no incentives offered. Responses were collected anonymously to ensure confidentiality and minimise bias during data collection and analysis. Participants could review and change their answers before submission by navigating back through the survey. They were also free to skip specific questions or withdraw from the survey at any time without providing a reason.

### 2.6. Data analysis

Participants’ data were only included in the analysis if they completed the survey. The closed questions were analysed using descriptive statistics such as frequency distribution and percentages. The open-ended questions were analysed using qualitative content analysis [16]. This analytic approach is particularly suitable for open-ended survey questions, where keywords or short phrases are identified, grouped and quantified to capture patterns in participant responses [17]. Given that most responses consisted of single words or short phrases, content analysis was considered appropriate, as it allows systematic classification and frequency-based interpretation of textual data. Responses were de-identified, coded and imported into Excel for analysis. The lead author (TZ) independently read and re-read the text to identify keywords from the responses. Subsequently, the data were thematically organised, and the main themes were identified, grouped and quantified using frequency counts, with assistance from two research colleagues experienced in qualitative analysis.

## 3. Result

### 3.1. Baseline demographics

The baseline characteristics of the 85 participating primary care healthcare professionals, including gender, age, profession and years of clinical experience, are summarised in Table 1. Among the healthcare professionals, the gender distribution was 46.0% male and 54.0% female, with a mean age of 44.1 ± 10.4 years. Healthcare professionals constituted GPs (37.6%) and physiotherapists (62.4%). In terms of clinical experience, 51.8% of healthcare professionals had more than 10 years of practice, while 48.2% had less than 10 years of practice.

**Table 1.** Baseline characteristics of primary care healthcare professionals (*n* = 85).

Characteristics	Categories	n (%)
Age, mean ± *SD*	–	44.1 ± 10.4
Gender, *n* (%)	Male/female	39(46.0) / 46(54.0)
Professional role	GP / physiotherapist	32(37.6) / 53(62.4)
Years of experience	0–5 / 6–10 / 11–20 / >20	16(18.8) / 25(29.4) / 27(31.8) / 17(20.0)

*Notes:* GP: general practitioner. Values are reported as mean ± standard deviation or frequency (percentage) as appropriate.

The baseline characteristics of the 138 participants with CLBP, including gender, age, ethnicity and pain duration, are summarised in Table 2. The gender distribution was skewed towards females (58.0%), with males comprising 42.0% of the sample. The mean age of participants was 45.3 ± 22.0 years. The ethnic composition was predominantly White (72.5%), followed by Asian (14.5%), Black (10.9%) and Other (2.2%) groups. Regarding pain duration, 76.1% of patients reported experiencing moderate-duration pain (3–6 months) and 23.9% reported chronic pain lasting more than six months, in line with the study’s eligibility criteria of at least three months’ duration.

**Table 2.** Baseline characteristics of people with CLBP (*n* = 138).

Characteristics	Categories	*n* (%)
Age, mean ± *SD*	–	45.3 ± 22.0
Gender	Male/female	58 (42.0) / 80 (58.0)
Ethnicity	White/Asian/Black/other	100 (72.5) / 20 (14.5) / 15 (10.9) / 3 (2.2)
Pain duration	Moderate (3–6 months)/chronic (>6 months)	105 (76.1) / 33 (23.9)

*Notes:* CLBP: chronic low back pain. Values are reported as mean ± standard deviation or frequency (percentage) as appropriate.

### 3.2. Type, delivery mode and perceived effectiveness of management provided by healthcare professionals

Tables 3 and 4 summarise the type, frequency and perceived effectiveness of the management approaches reported by GPs and physiotherapists, respectively, in managing CLBP in primary care. These data were obtained from open-text responses, where participants described the interventions they typically used and commented on their perceived effectiveness.

**Table 3.** Proportion of GPs using management approaches for CLBP and its perceived effectiveness.

Management approach	*N* (%) of GPs (total number = 32)	Perceived as helpful	Perceived as unhelpful
Exercise	28 (87.5%)	23	5
Pain education	6 (18.8%)	3	3
Active lifestyle	5 (15.6%)	5	0
Analgesic	27 (84.4%)	13	14
Psychological intervention	6 (18.8%)	5	1
Ergonomic advice	9 (28.1%)	8	1
Physiotherapy	20 (62.5%)	10	10

*Note*s: GPs: general practitioners; perceived effectiveness percentages are calculated based on the number of healthcare professionals who reported using each specific intervention.

**Table 4.** Proportion of physiotherapists using management approaches for CLBP and its perceived effectiveness.

Management approach	*N* (%) of physiotherapists (total number = 53)	Perceived as helpful	Perceived as unhelpful
Exercise	52 (98.1%)	51	1
Pain education	39 (73.6%)	37	2
Active lifestyle	25 (47.2%)	20	5
Analgesic	3 (5.7%)	1	2
Psychological intervention	14 (26.4%)	11	3
Ergonomic advice	11 (20.8%)	9	2
Manual therapy	15 (28.3%)	12	3
Self-management	10 (18.9%)	8	2
Electrotherapy	10 (18.9%)	7	3

*Notes:* Perceived effectiveness percentages are calculated based on the number of healthcare professionals who reported using each specific intervention. In the context of this study, manual therapy refers to hands-on techniques such as joint mobilization, manipulation and soft tissue techniques performed by trained healthcare professionals to alleviate pain and improve function.

Among GPs (Table 3), the most frequently reported management approaches were exercise (87.5%, 28/32), analgesic prescription (84.4%, 27/32), physiotherapy referral (62.5%, 20/32) and ergonomic advice (28.1%, 9/32). Physiotherapists (Table 4) most commonly reported exercise (98.1%, 52/53), pain education (73.6%, 39/53), active lifestyle advice (47.2%, 25/53) and manual therapy (28.3%, 15/53).

For both groups, most clinicians who reported using a given intervention perceived it to be helpful. Specifically, among GPs, exercise was considered helpful by 82.1% (23/28), active lifestyle advice by 100% (5/5), psychological intervention by 83.3% (5/6) and ergonomic advice by 88.9% (8/9). Pain education was perceived as helpful by 50% (3/6) and physiotherapy referral by 50% (10/20). In contrast, only 48.1% (13/27) of GPs who prescribed analgesics considered them helpful. Among physiotherapists, exercise was considered helpful by 98.1% (51/52), pain education by 94.9% (37/39), active lifestyle advice by 80% (20/25), psychological intervention by 78.6% (11/14), ergonomic advice by 81.8% (9/11), manual therapy by 80% (12/15), self-management by 80% (8/10) and electrotherapy by 70% (7/10).

A total of 35 (41.2%) of the 85 surveyed healthcare professionals described how they delivered their management strategies. Of these, 25 professionals delivered advice face-to-face in a one-on-one setting, 5 provided information remotely *via* printed materials or online resources and another 5 used a hybrid model combining face-to-face and remote methods.

### 3.3. Type and helpfulness of current treatments reported by people with CLBP

Table 5 summarises the type and helpfulness of interventions adopted by people with CLBP, based on responses to open-text questions. People reported receiving multiple treatments for their CLBP; most (*n* = 62, 44.9%) had used analgesics. A smaller proportion of participants reported being prescribed exercise (*n* = 38, 27.5%), physiotherapy (*n* = 25, 18.1%), manual therapy (*n* = 17, 12.3%), surgery (*n* = 17, 12.3%), electrotherapy (*n* = 17, 12.3%) and spinal injection (no clarity on the type of injection adopted) (*n* = 13, 9.4%).

**Table 5.** Proportion of people with CLBP adopting treatments and their perceived effectiveness.

Current treatment	*N* (%) people with CLBP (total number = 138)	Perceived as helpful	Perceived as unhelpful
Analgesics	62 (44.9%)	38	24
Exercise	38 (27.5%)	30	8
Physiotherapy	25 (18.1%)	21	4
Manual therapy	17 (12.3%)	14	3
Surgery	17 (12.3%)	15	2
Electrotherapy	17 (12.3%)	14	3
Spinal injection	13 (9.4%)	10	3

*Notes:* CLBP: chronic low back pain. Perceived effectiveness percentages are calculated based on the number of people who reported using each treatment. In the context of this study, manual therapy refers to hands-on techniques such as joint mobilization, manipulation and soft tissue techniques performed by trained healthcare professionals to alleviate pain and improve function.

Most people with CLBP who used specific treatments reported them to be effective. Among those who used analgesics, 60.8% (38/62) found them helpful; 77.8% (30/38) of patients engaging in exercise reported benefits; and 83.3% (21/25) of those receiving physiotherapy noted positive outcomes. Similarly, 80% (14/17) of patients who tried manual therapy, 90% (15/17) who underwent surgery, 85% (14/17) who adopted electrotherapy and 80% (10/13) who had spinal injections considered these interventions helpful.

### 3.4. Treatment goals

Treatment goals are outcomes that healthcare professionals or people with CLBP believe are important to achieve in managing lower back pain. Table 6 outlines the domains of care considered important by GPs for people with CLBP; these included functional improvement (*n* = 30, 94%), pain reduction (*n* = 26, 81%), anxiety and depression (*n* = 29, 91%), daily physical activity (*n* = 25, 78%), health-related quality of life (*n* = 24, 75%) and pain medication use (*n* = 25, 78%).

**Table 6.** Domains of care considered important by GPs for people with CLBP.

Objective	*N* (%) of GPs (total number = 32)
Functional improvement	30 (94%)
Pain reduction	26 (81%)
Health-related quality of life	24 (75%)
Anxiety and depression	29 (91%)
Daily physical activity	25(78%)
Pain medication use	25 (78%)

*Note:* GPs: general practitioners.

Table 7 presents the domains of care considered important by physiotherapists. The majority highlighted pain reduction (*n* = 48, 91%), anxiety and depression (*n* = 46, 87%), functional improvement (*n* = 45, 85%), daily physical activity (*n* = 44, 83%), health-related quality of life (*n* = 37, 70%) and pain medication use (*n* = 38, 72%).

**Table 7.** Domains of care considered important by physiotherapists for people with CLBP.

Objective	*N* (%) of physiotherapist (total number = 53)
Functional improvement	45 (85%)
Pain reduction	48 (91%)
Health-related quality of life	37 (70%)
Anxiety and depression	46 (87%)
Daily physical activity	44 (83%)
Pain medication use	38 (72%)

In contrast, Table 8 presents the treatment goals as perceived by people with CLBP. The most commonly reported goals were pain relief (*n* = 72, 52%) and getting back to daily physical activity (*n* = 46, 33%), followed by less pain medication use (*n* = 17, 12%), getting back to work (*n* = 12, 9%), improving mood (*n* = 12, 9%) and experiencing fewer back pain episodes (*n* = 4, 3%).

**Table 8.** Treatment goals by proportion of people with CLBP who considered them important.

Objective	*N* (%) of people with CLBP (total number = 138)
Pain relief	72 (52%)
Getting back to daily physical activity	46 (33%)
Getting back to work	12 (9%)
Less pain medication use	17 (12%)
Improving mood	12 (9%)
Less back pain episode	4 (3%)

## 4. Discussion

### 4.1. Management of healthcare professionals in primary care practice

#### 4.1.1. Evidence-based clinical management

This study suggests that most healthcare professionals used the following interventions in the clinical management of CLBP: exercise, pain education, active lifestyle, analgesic and psychological interventions. Our survey findings mirror the recommendations of the current NICE guidelines [7]. A recent review summarised key messages from recent clinical guidelines on LBP management published in the United States, Belgium, Denmark and the United Kingdom. It also found that advice, reassurance, self-management, medications and physical and psychological therapies are often first-line or second-line care for CLBP [18]. This may indicate that current management in primary care is generally aligned with clinical guidelines and evidence.

However, when disaggregating the results by profession, clear differences emerged between the medical and rehabilitation perspectives. GPs, reflecting a medical approach, more frequently recommended pharmacological interventions such as analgesics (84.4%) alongside exercise and physiotherapy referral. In contrast, physiotherapists, adopting a rehabilitation perspective, predominantly prescribed exercise (98%), pain education (73.6%) and active lifestyle advice (47.2%), with limited use of medication. These variations reflect the professional scope and training of each group but also suggest that patients may receive differing emphases in care depending on which provider they first consult.

The survey also noted that passive interventions aimed at reducing pain and muscle tension (e.g. rest, electrotherapy and manual techniques) are less frequently recommended in primary clinical care. In contrast, active interventions (e.g. exercise, education and psychological interventions) aimed at improving strength, function and awareness and reducing psychological effects accounted for the majority of interventions. These observations appear to align with the results presented in our previous study and are consistent with recommendations of a recently published Lancet back pain series [2,3]. The series advocated self-management, physical and psychological therapies and some forms of complementary medicine to manage CLBP, with less emphasis on hands-on treatment, electrotherapy and surgical options [3]. This finding is consistent with recent research suggesting that CLBP management in primary care may be shifting from a reactive to a proactive approach to LBP care over the past decade [19,20].

#### 4.1.2. Multidisciplinary biopsychological model

Our findings indicate that current primary care practices often rely on exercise interventions, patient education, or medication and very few reported involving social and psychological interventions as a holistic approach to manage CLBP. Access to psychological services was not specifically assessed in this study and the limited implementation of biopsychosocial approaches among primary care clinicians may partly reflect service availability constraints rather than a lack of awareness or willingness. Considering the complex nature of CLBP, a holistic biopsychological approach that integrates physical, psychological and social factors can be more effective in managing and relieving CLBP [21]. With pain and impaired functioning, people with CLBP frequently experience anxiety and depression, as well as effects on social, recreational and work-life [22]. There is evidence that psychological and social factors may play a role in developing and maintaining pain and disability [18,23]. The biopsychosocial model is also endorsed in many recently published clinical practice guidelines for the treatment of CLBP: the NICE guideline [24], the ACP guideline [25], the KCE guideline [26] and the TOP guideline [27]. A recent systematic review has highlighted the promising effects of multidisciplinary biopsychosocial interventions for patients suffering from CLBP [22]. A 2014 Cochrane review also showed that multidisciplinary biopsychosocial rehabilitation programmes are effective in reducing pain disability and improving work status in CLBP [23]. The biopsychological model of care is also rarely considered in healthcare practices in other regions. Indeed, only 8.4% of patients with LBP in the USA were prescribed cognitive behavioural therapy [28]. In Iran, managing patients with LBP is predominantly based on a traditional biomedical model and therapeutic interventions based on a biopsychosocial approach are rare [29].

Importantly, according to the NICE guidelines, a tiered or triaging approach is recommended for multidisciplinary team (MDT) involvement [7]. This means that additional MDT members, such as psychologists or social workers, are engaged based on patient complexity, severity and individual biopsychosocial needs, ensuring tailored and efficient care delivery rather than universal application across all patients [30]. To improve care for people with CLBP, clinicians may need targeted training to enhance their understanding of psychosocial pain mechanisms [31]. Alternatively, new models of care delivery may be developed to ensure patients with complex needs can access appropriate biopsychosocial support. These strategies could potentially contribute to closing the gap between evidence-based guidelines and real-world practice, ultimately leading to improved outcomes for people living with CLBP.

While the traditional BPS model provides a comprehensive framework, its clinical application has often been criticised as reductionist and fragmented. The biopsychosocial-enactive (BPS-E) model, proposed as an evolution of the BPS approach, places greater emphasis on patients’ embodied experiences, contextual factors and active meaning-making processes in pain. Integrating BPS-E principles may offer a more holistic and person-centred direction for CLBP management, potentially addressing gaps between clinical guidelines and lived experiences of patients [32].

### 4.2. Adherence of people with CLBP in the primary care practice

#### 4.2.1. Patient education

In this survey, all healthcare professionals recommended, to varying degrees, that people with CLBP in primary care engage in exercise, maintain an active lifestyle and adopt education about their back pain. This approach is also supported by a national survey in Ireland, which showed that the most common treatments used by physiotherapists for patients with CLBP were advice and exercise [33].

Despite patient education being provided by most healthcare professionals, very few participants with CLBP mentioned its use or being provided with such information. This divergence of experience reported by our clinicians and CLBP population is of interest and may reflect our CLBP patient’s beliefs or perception on education and what it is. Patient education means advising the patient on how to prevent LBP from recurring in the future, providing exercises and advice and encouraging patients to accept overall responsibility for their condition. A comparative study examining the perceptions of patients and GPs regarding patient education for LBP revealed that patients frequently lack the motivation necessary to adopt prevention-related skills or behaviours and may not be prepared to take responsibility for their LBP management [34]. This finding suggests that patients might not recognise education as a treatment for back pain, possibly perceiving the information provided as simply part of their consultation rather than central to their care. Consequently, it is crucial for healthcare providers to highlight the significance and integral role of educational interventions in the management of CLBP during clinical practice.

Another potential reason relates to the time constraints frequently experienced in clinical practice [35]. In the survey, some clinicians expressed that they do not have enough time to educate their patients appropriately in primary care. Lack of time will lead to barriers to communication between clinicians and patients [36] and may prevent the successful delivery of patient education and advice [37]. A systematic review showed that pain education for patients with LBP usually requires up to 2 h of contact with a trained health practitioner [38]. Other healthcare professionals are also constrained, a 2021 survey of current practice in Germany showed that the typical physiotherapy session for patients with musculoskeletal disorders lasts approximately 15-20 min, which poses a significant structural barrier to getting the message across [39]. Another study also reported that treating patients with LBP in a typical 15-minute primary care patient visit setting was a significant challenge [40].

#### 4.2.2. Pharmacological interventions

In the current survey of healthcare professionals, over 80% of GPs reported routinely prescribing medication to patients with CLBP, such as NSAIDs and analgesia. This is inconsistent with current clinical guidelines suggesting that pharmacological interventions should only be considered if there is an inadequate response to non-pharmacological interventions [24,25,41]. One possible explanation is the challenges clinicians encounter in achieving effective doctor-patient communication [42]. Patients often interpret the act of being prescribed medication or receiving passive therapies as a sign of being heard. Thus, patients often seek tangible forms of treatment from clinicians, such as medications, which can offer them a sense of being acknowledged and properly cared for [43]. Another reason may be that many healthcare systems struggle to deliver non-pharmacological care, particularly physical and psychological therapies and thus preferentially fund interventions such as surgery and prescribed medication [3]. Similarly, the people with CLBP surveyed in our study noted medication as essential to their care. Almost half (*n* = 74, 44.8%) of people with CLBP took medicine for their back pain. Such a trend also applies to high-income and low-income settings, with a general practice survey in Australia between 2000 and 2010 showing that 64.5% of patients adopted prescription medication for a new episode of LBP at their first visit [44]. Similarly, 90% of primary care patients in South Africa adopted analgesics as the only form of treatment for their back pain [45]. Treatment preferences and beliefs on pain medication of people with CLBP may be another contributing factor. A systematic scoping review conducted by Chou and his colleagues highlighted the concern for symptoms associated with back pain and the strong desire for pain control in patients with LBP, which may be highly correlated with the use of medications [46]. A cross-sectional study conducted in Germany found that patients often believe that medication provides faster and better pain relief than non-pharmacological treatments [47].

In terms of the helpfulness of medication, 50% (*n* = 15) of healthcare professionals claimed it was not helpful in the management of CLBP and 39% (*n* = 29) of people with CLBP also reported it was not helpful. This may reflect the current controversial evidence concerning pharmacological use in managing LBP. Some studies have demonstrated that NSAIDs, paracetamol, opioids, muscle relaxants and antidepressants only have a limited effect in treating non-specific LBP [48–50]. The reasons for negativity around medications may also stem from reported adverse events connected to these drugs, including the risk of addiction, drowsiness, nausea and dizziness, especially when taken over more extended periods [48,51].

### 4.3. Expectations for back pain treatment and its outcome

This study has observed a disconnect between what people with CLBP find useful and what clinicians find useful in managing CLBP. Most clinicians reported an active treatment approach, including exercise, pain education, active lifestyle, psychological interventions, ergonomic advice and self-management. This suggests that clinicians are endeavouring to encourage patients to self-manage their condition, promote behaviour changes and resume all functional activities, including work. However, patient-reported approaches focus primarily on passive interventions, including medication, manual therapy, electrotherapy and surgery. There is a tendency for patients to want to ‘get rid’ of symptoms and to expect someone else to be responsible for their condition. Research indicates that patients often have expectations of complete or nearly complete pain relief, which may not always align with the realistic outcomes of treatments [8,52]. In the study titled ‘Meeting Patients’ Expectations’, Engel points out that achieving a 50% reduction in pain is often deemed insufficient by patients, leading to feelings of disappointment [53]. A study by Weiner and colleagues also showed a lack of consensus between patients and clinicians on what to expect from chronic back pain management [54]. A recent narrative review highlights how patient expectations—such as desire for personalised therapy, validation, longer follow-up and meaningful clinician communication—are associated with better outcomes in conservative CLBP management, illustrating the importance of aligning care strategies with these expectations [55].

This difference is also reflected in clinicians’ and patients’ attitudes towards treatment goals. Most healthcare professionals highlighted the following areas: functional disability, pain intensity, health-related quality of life, anxiety and depression, getting back to daily physical activity and pain medication use, as necessary goals to address with CLBP. This implies that clinicians may consider a wide range of domains, from clinical, health status and psychological measurements to measures of physical function when managing CLBP. These results align closely with an earlier systematic review that emphasised the importance of a holistic approach to evaluating CLBP treatment [56]. However, from the perspective of people with CLBP, the priority is still being placed on pain relief and getting back to daily physical activity. This reflects that the treatment outcomes of greatest concern to people with CLBP remain focused on pain and function.

### 4.4. Person-centred care

This survey suggested differences between guideline-based primary care practice and the treatments people with CLBP reported using and finding helpful. Guideline-based treatments prescribed by healthcare professionals, such as patient education and psychosocial interventions, are not always aligned with those treatments adopted by people with CLBP. Conversely, some treatments, including pharmacological therapies and manual therapy, which are recommended as adjunctive or conditional interventions in clinical guidelines, are often perceived by patients as highly beneficial. These discrepancies may partly reflect that healthcare practitioners do not always fully incorporate or align patient preferences and expectations with guideline recommendations during clinical decision-making. Consequently, the differences in treatment approaches between healthcare professionals and patients highlight potential gaps between guideline-based management and truly person-centred care. A study of Australian GPs reported that recommendations in CPGs are sometimes inappropriate because patients’ priorities and needs can be overlooked [57]. Healthcare practitioners need to understand individual patients’ needs and develop personalised treatments to provide more high-value care for CLBP. A qualitative study also suggested that patients value being listened to, trusted and treated as individuals, so that care can better meet their needs [58].

According to the Evidence-Based Medicine (EBM) model [59], primary care practitioners should incorporate and implement these three elements in managing CLBP: carefully considering patient preferences and expectations, guided by evidence-based clinical guidelines and underpinned by clinical reasoning. A recent review of EBM by Djulbegovic and Guyatt [60], remind us that although the original focus of EBM was to educate clinicians on how to understand and use published evidence to optimize clinical care, EBM has now progressed to recognise the limitations of evidence alone and has increasingly stressed the need to combine critical appraisal of the evidence with patient’s values and preferences through shared decision-making. This suggests that external guidelines need to be integrated with individual clinical expertise in deciding whether and how it matches the patient’s clinical state, predicament and preferences, and thus whether interventions should be applied [61]. However, consider that discrepancies between current patient preferences and the recommendations of evidence-based clinical guidelines. This highlights a potential ongoing challenge that healthcare providers may face in aligning patient expectations with the realities of CLBP management strategies. To navigate this complexity, future clinical practices will need to evolve, emphasising the importance of healthcare providers actively engaging in strategies to reconcile these differences. Through enhanced communication, shared decision-making and individualized care planning, there is an opportunity to bridge the gap between patient desires and the practical application of clinical evidence and expertise to deliver person-centred care.

### 4.5. Strengths and limitations

A notable strength of this study is its aim to explore differing perspectives and perceptual gaps in the management of CLBP between healthcare professionals and people with CLBP within UK primary care. By adopting a mixed-methods design, the study combines both quantitative patterns and qualitative insights, offering a nuanced understanding of how current clinical practice aligns—or diverges—from patient experiences and expectations. This dual-perspective approach, together with references to international guidelines, contributes to a more contextually grounded understanding of clinical practice and may offer insights of practical relevance. Another key strength is that the results for healthcare professionals are presented separately for general practitioners and physiotherapists. This approach allows for a direct comparison of management perspectives from ‘medical’ and ‘rehabilitation’ viewpoints, highlighting profession-specific priorities and providing richer, more actionable insights into multidisciplinary care for CLBP.

However, some limitations should be acknowledged. The relatively small and narrowly defined sample may limit the generalisability of results. Recruitment was further affected by ongoing post-pandemic constraints, such as workforce shortages and limited availability. Opportunistic sampling also raises the risk of selection bias, and reliance on self-reported data may have introduced recall bias. Finally, the cross-sectional design captures only a single time point, limiting the ability to infer causality or observe changes over time. Future studies should aim for broader, more representative samples, clearer inclusion controls and longitudinal approaches to enhance the robustness and applicability of findings.

### 4.6. Implications for clinical practice and future research

This study revealed gaps between evidence-based guidelines and the treatments reported by people with CLBP in primary care, particularly regarding the limited uptake of education, active interventions and self-management strategies. Disaggregated results further showed profession-specific patterns: GPs, reflecting a medical perspective, more frequently prioritised pharmacological management alongside some active strategies, whereas physiotherapists, reflecting a rehabilitation perspective, focused more heavily on exercise, education and active lifestyle promotion. These findings highlight the need for improved communication between healthcare professionals and patients, as well as greater integration of medical and rehabilitation perspectives through interdisciplinary collaboration, to ensure that care addresses both symptom control and functional restoration.

Addressing these gaps will require ongoing clinician training in biopsychosocial pain management, stronger emphasis on shared decision-making and person-centred care and system-level reforms to overcome barriers such as limited access to psychological services and short consultation times. Future research should explore how best to incorporate patient values and expectations into guideline implementation, evaluate the long-term outcomes of multidisciplinary interventions across diverse settings and draw on larger, more representative samples to inform policy changes that promote high-value, patient-centred care for CLBP.

## 5. Conclusion

This study provides contemporary insight into the clinical practices of GPs and physiotherapists managing CLBP and the lived experiences of individuals with CLBP in UK primary care. While the reported practices of healthcare professionals generally align with clinical guidelines, there are notable gaps between recommended care and what patients report receiving or choosing to adopt. These findings highlight a disconnect between healthcare provider delivery and patient uptake, influenced by factors such as patient preferences, perceived effectiveness and access. This study fills an important gap by comparing perspectives from both sides of the clinical encounter and underscores the need for improved strategies in shared decision-making and patient engagement to better align care with both evidence and individual needs.

## Supplementary Material

Appendix D.docx

Appendix A.docx

Appendix C.docx

Appendix B.docx

## Data Availability

The data sets generated and analysed during this study are available from the corresponding author on reasonable request.

## Transparency

The authors confirm that the submitted manuscript is an honest, accurate and transparent account of the study being reported. No important aspects of the study have been omitted.
