# Supplementary material for: Perception gaps between healthcare professionals and people with CLBP: an online survey of current primary care management practices in the United Kingdom
Source: Ann Med. 2025 Sep 17;57(1):2553216. doi: 10.1080/07853890.2025.2553216 (PMC12444962; doi:10.1080/07853890.2025.2553216)
Supplement: Appendix D.docx [file IANN_A_2553216_SM1429.docx]

Appendix D. Survey questionnaire for people with experience of low back pain


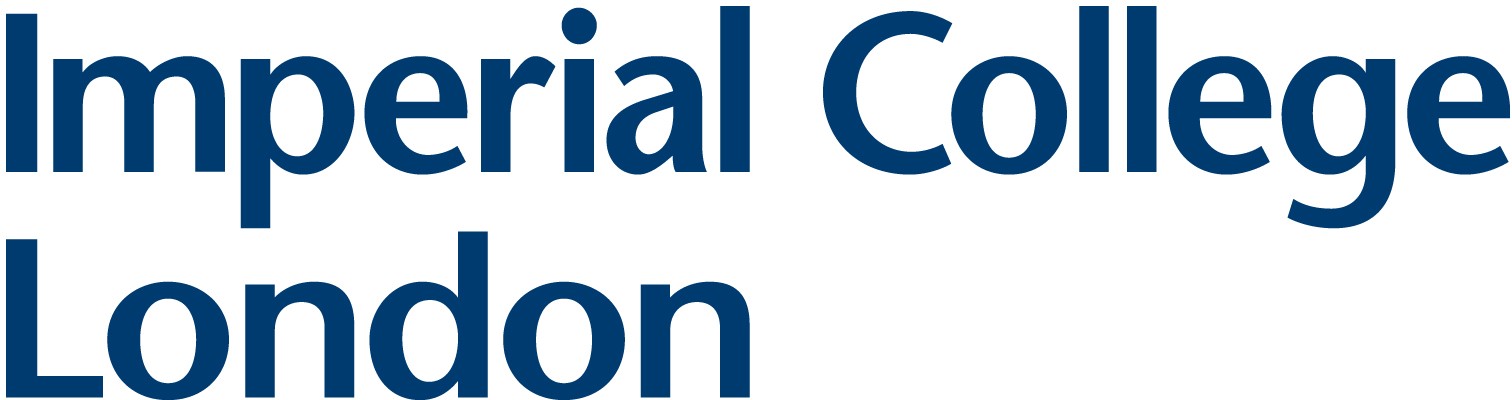


1. What is your age?





1. What is your gender?

- Male
- Female
- Prefer not to say

1. What is your race?


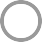
 White
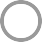
 Asian
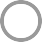
 Black


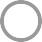
 Other (please specify)

1. How long have you had your back pain?


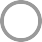
 0-3 months


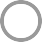
 3-6 months


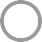
 Over 6 months

1. What treatment have you been given for your back pain?
2. Do you think the treatment helped? Can you tell us why


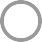
 Yes


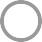
 No

1. What sort of treatment do you think would help your back pain?
2. What things would you help you know that you were getting better? Please let us know why


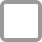
 Pain relief


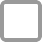
 Getting back to normal physical activity


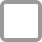
 Getting back to work


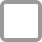
 Less pain medication use


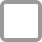
 Improving mood


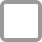
 Other (please specify)

1. Are there any further comments you’d like to make?

We'd like to develop a self-management app to help you manage your back pain. If you would be interested in discussing your experiences with us and/or helping us with a workshop to develop this tool, please leave your contact details (First name and Email address) below for further information. Your details would only be used to contact you.
