# Supplementary material for: Perception gaps between healthcare professionals and people with CLBP: an online survey of current primary care management practices in the United Kingdom
Source: Ann Med. 2025 Sep 17;57(1):2553216. doi: 10.1080/07853890.2025.2553216 (PMC12444962; doi:10.1080/07853890.2025.2553216)
Supplement: Appendix C.docx [file IANN_A_2553216_SM1427.docx]

Appendix C. Survey questionnaire for healthcare professionals


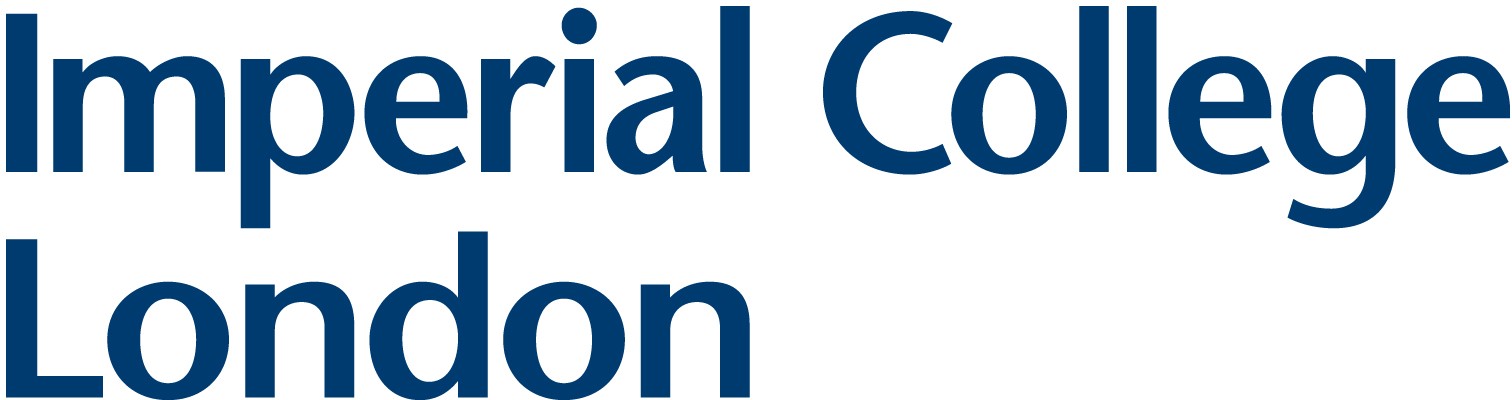


1. What is your age?





1. What is your gender?

- Male
- Female
- Prefer not to say

1. What is your role in primary care?


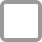
 Physiotherapist
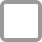
 Pharmacist


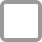
 GP


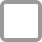
 Nurse


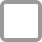
 Healthcare assistant
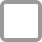
 Osteopath


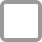
 Psychologist


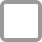
 OtherOther (please specify)

1. How many years have you been in this role?

- 0 to 5 years
- 6 to 10 years
- 11 to 20 years
- Over 20 years

1. What advice do you give to your patients with Chronic Low Back Pain (CLBP)? And how is it delivered?
2. What treatment do you give to your patients with CLBP? And how is it delivered?
3. Do you think the advice you give to patients is effective? Please let us know why

- Yes
- No

1. Do you think the treatment you give to patients is effective? Please let us know why

- Yes
- No

1. What outcomes do you think are important to CLBP patients? Please specify why you select this option


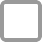
 Functional disability


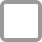
 Pain intensity


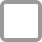
 Health-related quality of life


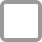
 Anxiety and Depression


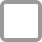
 Physical activity


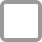
 Pain medication use


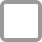
 Other (please specify)

1. Are there any further comments you’d like to make?

We'd like to develop a self-management app to help people with CLBP manage their back pain. If you would be interested in discussing your experiences with us and/or helping us with a workshop to develop this tool, please leave your contact details (First name and Email address) below for further information. Your details would only be used to contact you.
